# The Wounded, the Women of England, and the Voluntary Hospitals

**Published:** 1914-11-28

**Authors:** 


### The Hospital

The Workers' Newspaper of Administrative Medicine and Institutional Life, Administration, National Insurance and Health.

No. 1484, Vol. LVII. SATURDAY, NOVEMBER 28, 1914. PRICE ONE PENNY.

### THE WOUNDED, THE WOMEN OF ENGLAND, AND THE VOLUNTARY HOSPITALS.

In our issue of the 21st instant, in an article on "Duty-free Rectified Spirit in Hospitals," we produced evidence that there never has been a period when the British voluntary hospitals have rendered more useful and important national service than in the present crisis. Throughout the United Kingdom there has been a unanimous readiness on the part of hospital committees to place the greatest possible facilities at the disposal of the Government for the treatment of wounded sailors and soldiers, at a cost which must inevitably add to the burdens of institutional finance. Several hospitals have admitted would-be recruits rejected from the Service on account of physical disability, who have been restored to a condition of health which has enabled them to join the Colours. The Territorial hospitals are largely equipped from the staffs of the voluntary hospitals, and the requisite numbers of fully trained nurses have been mainly derived from the same institutions. In addition, fully trained nurses have been supplied to the Queen Alexandra Imperial Military Nursing Service, and resident medical officers of all grades have been given facilities to serve with the Imperial forces. All this spontaneous service has been rendered by the voluntary hospitals with the promptest good-will, and, although in some cases the Government have sanctioned a grant of a small daily payment averaging 3s., the balance of the cost of the treatment of wounded warriors over and above the Government grants has been and is considerable. It is of the first importance that the heavy outlay entailed upon the voluntary hospitals by placing some 10,000 beds at the disposal of the War Office for wounded soldiers, and the heavy drain thus made upon the resources of our voluntary hospitals, should be fully known to and appreciated by the public, and also by the Government.

There is another side to this question. The war has excited a keen desire in the minds of a multitude of people to render some personal service in the cause of the nation, and it is but just to say that the women of Great Britain have set the men a noble example in this respect. We hope the men will quickly awaken fully to a sense of their individual responsibility and come forward in increasing numbers to join the Army. Women are proverbially quicker to see things than men, for had the men displayed equal readiness with the women to do their duty in respect to this war, the time of its duration would have been shortened probably by at least twelve months. If, therefore, we can excite the whole-hearted interest of the women of England in the treatment of wounded sailors and soldiers in our voluntary hospitals, they will utilise their energies with results fruitful for good to the wounded, the voluntary hospitals, and themselves.

We are gratefully conscious of the keenness of our women to be up and doing, so that they may worthily take their part in helping the wounded and the nation during the war. We would especially urge upon every woman, who is keen to render personal service which shall prove completely effective in results, to realise the immense help which has been extended to the wounded by the voluntary hospitals throughout the country. If every woman could be brought to apprehend the actual value to the nation of this voluntary hospital service for the wounded, she would certainly gladly avail herself of the opportunity thus offered to share in that splendid work. Dependent as the hospitals are for their maintenance mainly upon voluntary contributions, the heavy extra charges due to the war are calculated to render it essential that many additional givers, especially of small sums, shall be recruited in support of the voluntary hospitals. King Edward in his appeal to the nation, when Prince of Wales, on the occasion of Queen Victoria's Diamond Jubilee, recognised the immense value of small gifts, and created by Royal Charter the League of Mercy to enable everyone to have an opportunity to render personal service and to secure to every giver of small sums, however humble, a guarantee that each gift would be conveyed to the hospitals' exchequer without any deduction whatever.

The war is likely to continue for three years at least, and if our women would take into their hands the receipts of the contributions from the small givers by becoming members of the League of

Mercy, they would attract to themselves the gratitude of all sick people, and make their names memorable in the history of the nation when the war is over. We shall be glad if our readers will bring this article to the notice of the women of their family and their friends, with the assurance that if they will communicate with the Editor of The Hospital he will gladly put them in the way of joining the Women's Hospital Brigade. They can so make their personal service for the sick and wounded more fruitful in result than any other work can secure to them. **We must recruit 50,000 women for this Brigade** to make it adequate to defray the cost of treating the sick and wounded in the voluntary hospitals. Who will help? Or rather does any woman exist who will refuse to enlist in the Women's Voluntary Hospitals Brigade?

